# Targeting IgG Autoantibodies for Improved Cytotoxicity of Bactericidal Permeability Increasing Protein in Cystic Fibrosis

**DOI:** 10.3389/fphar.2020.01098

**Published:** 2020-07-17

**Authors:** Karen McQuillan, Fatma Gargoum, Mark P. Murphy, Oliver J. McElvaney, Noel G. McElvaney, Emer P. Reeves

**Affiliations:** Irish Centre for Genetic Lung Disease, Department of Medicine, Royal College of Surgeons in Ireland, Education and Research Centre, Beaumont Hospital, Dublin, Ireland

**Keywords:** cystic fibrosis, neutrophils, bactericidal permeability increasing protein, IgG class autoantibodies, *Pseudomonas aeruginosa*

## Abstract

In people with cystic fibrosis (PWCF), inflammation with concurrent infection occurs from a young age and significantly influences lung disease progression. Studies indicate that neutrophils are important effector cells in the pathogenesis of CF and in the development of anti-neutrophil cytoplasmic autoantibodies (ANCA). ANCA specific for bactericidal permeability increasing protein (BPI-ANCA) are detected in people with CF, and correlate with infection with *Pseudomonas aeruginosa*. The aim of this study was to determine the signaling mechanism leading to increased BPI release by CF neutrophils, while identifying IgG class BPI-ANCA in CF airways samples as the cause for impaired antimicrobial activity of BPI against *P. aeruginosa.* Plasma and/or bronchoalveolar lavage fluid (BAL) was collected from PWCF (n = 40), CF receiving ivacaftor therapy (n = 10), non-CF patient cohorts (n = 7) and healthy controls (n = 38). Plasma and BAL BPI and BPI-ANCA were measured by ELISA and GTP-bound Rac2 detected using an *in vitro* assay. The antibacterial effect of all treatments tested was determined by colony forming units enumeration. Levels of BPI are significantly increased in plasma (p = 0.007) and BALF (p < 0.0001) of PWCF. The signaling mechanism leading to increased degranulation and exocytosis of BPI by CF neutrophils (p = 0.02) involved enhancement of Rac2 GTP-loading (p = 0.03). The full-length BPI protein was detectable in all CF BAL samples and patients displayed ANCA with BPI specificity. IgG class autoantibodies were purified from CF BAL complexed to BPI (n=5), with IgG autoantibody cross-linking of antigen preventing BPI induced *P. aeruginosa* killing (p < 0.0001). Results indicate that the immune-mediated diminished antimicrobial defense, attributed to anti-BPI-IgG, necessitates the formation of a drug/immune complex intermediate that can maintain cytotoxic effects of BPI towards Gram-negative pathogens, with the potential to transform the current treatment of CF airways disease.

## Introduction

Cystic Fibrosis (CF) is an autosomal recessive pleiotropic disorder caused by mutations in the gene encoding the CF transmembrane conductance regulator (CFTR) chloride channel (Riordan et al., 1989; Rommens et al., 1989). Over 2000 CFTR mutations have been identified that result in disturbed synthesis or function of the CFTR protein, leading to the development of specialized therapies targeting the basic defect (Mcelvaney et al., 2018). The most common mutation is deletion of phenylalanine at position 508 (*Phe508del*) which is present in one or both alleles in approximately 90% of PWCF (Riordan, 2008). CF related symptoms, although variable from patient to patient, are present early in life and increase in severity with age, with respiratory manifestations impacting on morbidity and mortality. CFTR absence or malfunction causes defective ion transport, reduction in airway surface liquid (ASL) volumes and persistent mucus hypersecretion. Inflammation is further amplified by bacterial infections, initially *Haemophilus influenza* and *Staphylococcus aureus* in infants, and later *Pseudomonas aeruginosa*, with early eradication with anti-pseudomonal antibiotics demonstrating significant improvements in FEV_1_, reductions in exacerbations (Ramsey et al., 1999; Konstan et al., 2011) and in bacterial density (Mccoy et al., 2008). Accordingly, in the adult CF population, *S. aureus* infections without the presence of *P. aeruginosa*, is a marker of milder lung disease (Ahlgren et al., 2015). Conversely, a greater rate of decline of lung function has been recorded in young people with *P. aeruginosa* lung infection compared to infants with no infection detected (Pillarisetti et al., 2011).

Sustained neutrophil recruitment and neutrophil dominated inflammation are hallmarks of CF airways disease progression, yet paradoxically, recruited cells fail to kill invading bacteria, with chronic infection with mucoid, alginate-producing strains of *P. aeruginosa* a major cause of mortality. Primary granules of neutrophils contain a battery of antimicrobial mediators, including the potent cytotoxic bactericidal permeability increasing protein (BPI) that targets gram-negative bacteria such as *Pseudomonas*. BPI is a bipartite molecule with distinct domain functionality, involving anti-bacterial and endotoxin neutralization properties of *N*- and *C*-terminal domains, respectively. The remarkable ability of *P. aeruginosa* to colonize the CF airways despite the presence of BPI is not fully understood. A rise in anti-neutrophil cytoplasmic antibodies (ANCA) with BPI specificity have been identified in PWCF (Sediva et al., 1998; Mahadeva et al., 1999), varying from 17.9% to 83% positivity (Iwuji et al., 2019), with a strong correlation between IgA and IgG BPI-ANCA and reduced lung function recorded (Carlsson et al., 2007). Although anti-BPI IgA has been detected in CF bronchoalveolar lavage (BAL), strikingly, *in vitro* anti-BPI-IgG inhibits the antibiotic function of BPI (Schinke et al., 2004). This prompted us to investigate the *in vivo* concept that BPI present in CF airways is immune complexed to anti-BPI-IgG, and this direct interaction inhibits anti-*Pseudomonas* activity.

## Materials and Methods

### Chemicals and Reagents

All chemicals and reagents were of the highest purity available and were purchased from Sigma-Aldrich Ireland unless indicated otherwise.

### Study Design

PWCF were recruited from the Beaumont Hospital Cystic Fibrosis Clinic. Ethical approval was received from the Beaumont Hospital Ethics Board (REC reference 14/98) and informed consent obtained from all study participants. Demographic details of PWCF are listed in **Tables 1** and **2**. The CF-ABLE score is a predictive score for determining outcome in CF, validated against the CF registry of Ireland (Mccarthy et al., 2013). Scores range from 0 to 7, with a higher score predicting a worse outcome. The CF-ABLE score of recruited patients was 3.68 ± 2.11 (**Table 1**). To assess the effect of ivacaftor therapy on plasma levels of BPI autoantibodies, clinically stable CF patients, homozygous or heterozygous for the *Gly551Asp CFTR* variant, receiving 150 mg ivacaftor twice daily (n = 10) were recruited (**Table 2**). Details regarding healthy controls (HC), patients with non‐cystic fibrosis bronchiectasis (NCFB) or chronic obstructive pulmonary disease (COPD) are available in **Table 3**.

**Table 1:** Demographic details of PWCF recruited to this study.

Clinical demographic parameter	CF participants
Total number	28
Age in years	27.7 ± 5.02
Male	16
Female	12
Homozygous for *Phe508del*	11
At least one *Phe508del* copy	25
FEV_1_	42.01 ± 21.92
BMI	20.06 ± 2.55
CF-ABLE score	3.68 ± 2.11
*P. aeruginosa* colonization at time of sample	19
*P. aeruginosa* colonization within last 5 years	28

Data are presented as number or mean ± standard deviation.

Phe508del, CFTR gene mutation; FEV_1_, forced expiratory volume in 1 s (% predicted); BMI, body mass index (kg/m^2^); P. aeruginosa, Pseudomonas aeruginosa.

**Table 2:** Demographic details of the CF cohort receiving ivacaftor therapy.

Patient	CFTR mutation	FEV_1_Pre-therapy(% predicted)	FEV_1_Post-therapy(% predicted)
1	*Phe508del/Gly551Asp*	45	56
2	*Phe508del/Gly551Asp*	25	31
3	*Phe508del/Gly551Asp*	53	71
4	*Phe508del/Gly551Asp*	45	64
5	*Phe508del/Gly551Asp*	93	86
6	*Phe508del/Gly551Asp*	56	27
7	*Phe508del/Gly551Asp*	57	95
8	*Gly551Asp/G542X*	24	24
9	*Gly551Asp/G542X*	45	40
10	*Gly551Asp/Gly551Asp*	30	36

FEV_1_, forced expiratory volume in one second.

**Table 3:** Characteristics of healthy controls, patients with chronic obstructive pulmonary disease or patients with non‐cystic fibrosis bronchiectasis recruited to this study.

Clinical demographic parameter	NCFB	COPD	HC
Total number	5	2	38
Age in years	68.53 ± 11.4	48	32.38 ± 4.6
FEV_1_	50.66 ± 15.22	64.5	101.33 ± 7.6
BMI	28.31 ± 5.5	25.91	25.84 ± 2.16

Data are presented as number or mean ± standard deviation.

NCFB, non‐cystic fibrosis bronchiectasis; COPD, chronic obstructive pulmonary disease; HC, healthy control; FEV_1_, forced expiratory volume in one second; BMI, body mass index (kg/m^2^).

### Preparation of Blood and Airways Samples

Blood samples were collected in 7.5-ml heparinized S-monovette tubes (10 U/ml; Sarstedt, Germany) and centrifuged at 350×*g* for 5 min at room temperature. Plasma was aliquoted for immediate use or stored at -80°C. Peripheral blood neutrophils were isolated using a previously described method, (Reeves et al., 2002) with all steps performed at room temperature. Purity of isolated neutrophils was validated by flow cytometric analysis using a monoclonal antibody against CD16b (Saeki et al., 2009; Bergin et al., 2014) and neutrophil viability was assessed by trypan blue exclusion assays. Bronchoalveolar lavage (BAL) samples were obtained from PWCF (**Table 4**). BAL was performed and samples processed as previously published (Rennard et al., 1998). Briefly, 100 ml of buffered saline was instilled into the right middle lobe or lingula and harvested. BAL was filtered through sterile gauze and then centrifugation at 462 x *g* for 10 min at 4°C. Cell-free supernatants were aliquoted and stored at −80°C for subsequent analysis.

**Table 4:** Demographic details of PWCF who donated BAL and plasma samples for LPS experiments.

Clinical demographic parameter	CF cohort (*Phe508del*)
Total number	12
Age in years	29.6 ± 5.61
Male	6
Female	6
Homozygous for *Phe508del*	12
FEV_1_	45.71 ± 17.92
BMI	20.88 ± 2.61
CF-ABLE score	3.72 ± 2.11
*P. aeruginosa* colonization	12

Data are presented as number or mean ± standard deviation.

Phe508del, CFTR gene mutation; FEV_1_, forced expiratory volume in 1 s (% predicted); BMI, body mass index (kg/m^2^); P. aeruginosa, Pseudomonas aeruginosa.

### Gel Electrophoresis and Western Blot Analyses

Samples were subjected to SDS-PAGE under denaturing and non-denaturing conditions using the NativePAGE™ Novex^®^ Bis-Tris gel system (Invitrogen™) following the manufacturer’s instructions. After Electrophoresis, gels were stained with Coomassie^®^ Brilliant blue G250 for visualization of proteins or transferred to 0.2 µM PVDF membrane (Roche) by western blotting. Blots were incubated with 0.2 µg/ml mouse monoclonal anti-BPI specific antibody (Santa Cruz Biotech Inc.), 0.01 mM rabbit polyclonal anti-Rac2 specific antibody (Cell Signalling Technology- Rac 1/2/3 antibody) or 1.0 μg/ml of monoclonal anti-actin antibody (Millipore, UK). The secondary antibodies used were HRP-linked rabbit anti-mouse and goat anti-rabbit IgG (Cell Signalling Technology). Immunoreactivity was detected using Immobilon™ Western Chemiluminescent HRP- substrate (Millipore) solution and a G-Box Chemie XL (Syngene) and analyzed using GeneSnap and GeneTools software.

### Quantification of LPS, BPI, or BPI ANCA Levels

Lipopolysaccharide (LPS) was quantified in plasma and BAL of PWCF (**Table 4**) using an LPS ELISA (Cusabio: catalog number CSB-E09945h). BPI levels were quantified using the Human BPI ELISA kit (Hycult^®^ Biotech: catalog number HK314-01). Anti-BPI (IgG) levels were evaluated using the Human anti-BPI ELISA kit (Orgentec: catalog number ORG 523). Plate readings were recorded using a Spectra Max M3 plate reader. All ELISA experiments were undertaken in accordance with the manufacturer’s instructions.

### Neutrophil Rac2 Activation and Degranulation Assays

The Rac activation kit (Abcam: catalog number ab139586) was used to isolate active GTP-bound Rac from neutrophil lysates. This kit utilizes the selective interaction of the Cdc42/Rac interactive binding domain (CRIB) of the effector p21 activated kinase-1 (PAK-1) with the active RacGTP conformation. Anti-Rac2 antibody was used to detect total and active GTP-bound Rac2 in neutrophil lysates by Western blotting. For neutrophil degranulation assays, cells (1 x 10^7^/ml) were either unstimulated or stimulated with TNF-α (1 ng/ml) and fMLP (100 ng/ml) at 37°C and 100 µl aliquots removed at 0, 5, 10 or 20 min and added to 4 volumes of ice cold PBS containing protease inhibitors (Nα-tosyl-L-lysine chloromethyl ketone hydrochloride (10 μg/ml), phenylmethanesulfonyl fluoride (1 μg/ml), pepstatin A (10 μg/ml), and leupeptin (10 μg/ml)). Cell free supernatants were harvested following centrifugation at 500×*g* for 5 min at 4°C and analyzed for degranulated BPI by Western blotting. The use of equal cell numbers (2 × 10^7^/ml) in each reaction was demonstrated by identical immune band intensity of actin in Western blots of whole cell lysates of cells used per reaction.

### Purification of IgG BPI-ANCA

Starting BAL (100μl) supplemented with protease inhibitors (as listed above) was incubated with Protein G Sepharose 4 Fast Flow (GE Healthcare) (30μl packed bed) and gently rotated for 1 h at 4°C. Protein G Sepharose 4 Fast Flow comprises immobilized recombinant protein G that binds to the Fc region of IgG. Reactions were centrifuged at 12,000 x g for 1 min and the unbound supernatant harvested. The Protein G Sepharose pellet was washed x 6 in 1 ml pre-chilled PBS containing protease inhibitors before addition of 2× Laemmli Sample Buffer with or without 1 mM DTT. Samples were boiled at 98°C for 3 min and subjected to SDS-PAGE and stained with Coomassie blue to visualize proteins or Western blotted for BPI. In a subset of experiments, BPI autoantibodies were purified as previously described (Bergin et al., 2014), concentrated using an Amicon^®^ Ultra-30K Centrifugal filter device, and protein concentration determined using a Nanodrop™ 8000, prior to use in bactericidal assays.

### BPI Anti-*Pseudomonas* Assays

Luria-Bertani (LB) broth was inoculated with a single bacterial colony of *P. aeruginosa* strain PAO1 and incubated overnight at 220 rpm at 37°C (Brunswick™ Excels^®^ E25 shaker incubator). Bacteria were sub-cultured into fresh LB broth and incubated until mid-logarithmic phase was reached (Bergsson et al., 2009). After culturing the bacteria were washed x 3 in PBS and resuspended at a density of 1 x 10^8^/ml. For BPI bactericidal assays, PAO1 was re-suspended in 0, 1, 2.5, 5, 10, 20 or 40 µg/ml of human neutrophil purified BPI (Athens Research) in PBS pH 7.5 at 37°C. An aliquot was removed at 32 min into ice cold LB broth and samples were diluted and plated in triplicate on LB agar plates and incubated overnight at 37°C. Colony forming units (c.f.u.) were counted, and the percentage survival was quantified by setting the number of c.f.u. with no BPI at 100% survival. Experiments were repeated in PBS set at pH 5.5, 6.5 or 7.5 in the presence of 10 µg/ml BPI for 10 min. Alternatively, bacteria were suspended in PBS pH 7.5 with BPI (10 µg/ml) in the presence of glycosaminoglycans (GAG) (heparin sulphate, chondroitin sulphate and hyaluronic acid (1 mg/ml of each)) for 2, 4 or 8 min. BPI inhibitory assays employed a rabbit *N*-terminal directed anti-BPI antibody (Sigma Aldrich: catalog number B2188) or CF purified anti-BPI IgG. BPI (10 µg/ml) was incubated in the presence or absence of antibody (10 µg/ml) for 20 min prior to use.

### Statistical Analysis

All data were analyzed using GraphPad Prism version 7 (La Jolla, CA, USA). Unless stated otherwise, data are expressed as mean ± SEM and p values were determined by Students t-test. One-way or two-way ANOVA was used to determine statistical significance when comparing three or more groups tested with one or two factors, respectively. A p value of ≤ 0.05 was deemed statistically significant following Bonferroni or Tukey *post-hoc* multiple comparision tests as indicated.

## Results

### Elevated Levels of BPI Are Present in the Plasma and BAL of People With CF Colonized With *P. aeruginosa*

**Figure 1:**
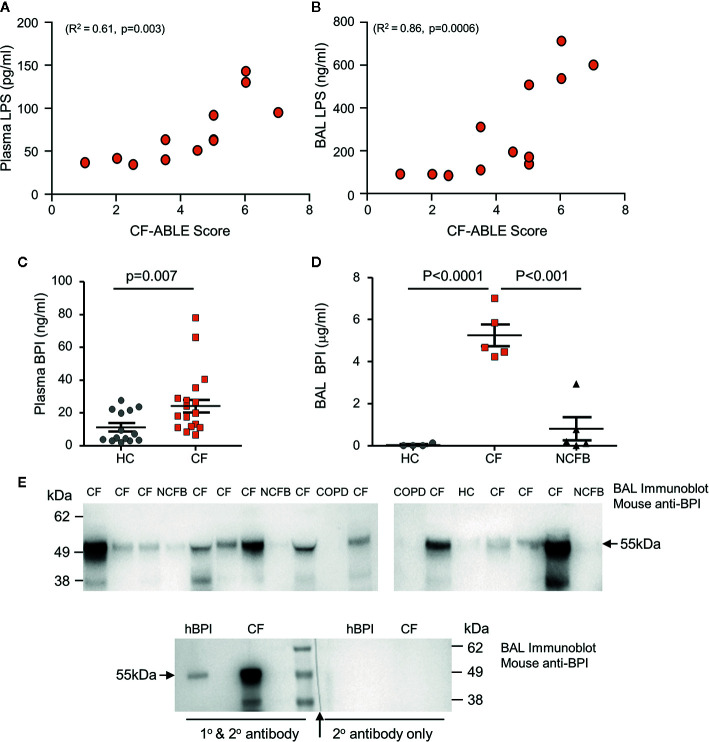
Elevated levels of BPI present in plasma and BAL of people with CF. **(A**, **B)** LPS levels in CF plasma and BAL correlated with CF-ABLE score. Quantification of an association between variables was achieved by Spearman correlation. **(C**, **D)** Comparative analysis of BPI present in plasma or BAL of *Phe508del* homozygous PWCF (CF), healthy controls (HC) or NCFB patients was performed by ELISA. **(C)** BPI levels were significantly increased in plasma of CF compared to HC (n=22 and n=14 subjects per group, respectively, p=0.007, Mann Whitney U-test). **(D)** BAL levels of BPI were significantly increased in CF (n=5) compared to HC (n=4) or NCFB patients (n=5) (p<0.0001, One-way ANOVA, followed by Bonferroni post-hoc test for selected groups). **(E)** BAL samples from HC, CF, NCFB or COPD were subjected to SDS-PAGE and Western blot analysis for BPI. An immuno-band of increased intensity for BPI was detected in CF BAL samples (top panels). Lower panel, a control immunoblot to ensure BPI specificity. The blot was halved, with one half probed with secondary antibody only with no BPI immune-bands visible (↑ indicates where blot was cut). Human BPI (hBPI) was used as a positive control. All measurements are means ± SEM from biological replicates.

PWCF were recruited from the Beaumont Hospital Cystic Fibrosis Clinic (**Table 4**). Patients had a mean % FEV_1_ of 45.71 ± 17.92 and CF-ABLE score of 3.72 ± 2.11. Patients were colonized with *P. aeruginosa* at the time of sampling or in the preceding 5 years. Confirming bacterial colonization, plasma (n=12, R^2 =^ 0.61, p=0.003) and BAL (n=12, R^2 =^ 0.86, p=0.0006) levels of LPS correlated directly with severity of CF airways disease, as determined by the CF-ABLE score (**Figures 1A, B**).

To investigate whether cytotoxicity of neutrophil BPI is limited against *P. aeruginosa* due to proteolysis, we first determined the levels and structural integrity of extracellular BPI protein *in vivo*. Plasma and BAL samples were obtained from PWCF, healthy controls (HC), or non-CF bronchiectasis (NCFB) patients for comparison. Quantification of BPI by ELISA revealed that plasma from PWCF contained significantly higher levels of soluble BPI than that measured in HC plasma (24.2 ± 3.9 ng/ml and 11.2 ± 2.6 ng/ml respectively, p=0.007) (**Figure 1C**). To further evaluate BPI levels *in vivo*, ELISA analysis of BAL was performed (**Figure 1D**). Results revealed that CF BAL had a greater than 100-fold increase in concentration of BPI when compared to HC BAL (4.6 ± 0.5 and 0.04 ± 0.03μg/ml respectively, p<0.0001) and a 5-fold increase compared to BAL from NCFB patients (0.8 ± 0.5μg/ml, p<0.001).

As increased levels of proteolytic activity can degrade structural and immune proteins present in the CF airways (Birrer et al., 1994; Margaroli et al., 2018), we next assessed the integrity of BPI and its ability to remain intact within the CF lung. Western blot was employed to detect BPI in CF BAL, and also NCFB, HC or COPD BAL samples for comparison. In CF BAL, although detected at various intensities, an immunoband for BPI of the correct molecular mass of 55 kDa was detected in all samples (**Figure 1E**). BPI was minimally detected in NCFB BAL and undetectable in COPD or HC BAL samples. The molecular mass of the protein and specificity of the BPI primary antibody employed was confirmed in control experiments employing secondary antibody only (**Figure 1E**, lower panel). Collectively these results indicate increased levels of extracellular BPI in the blood circulation and airways of PWCF, posing the question of whether CF neutrophils release significantly greater quantities of BPI, and the mechanism thereof.

### Increased Rac2 Activation in CF Neutrophils

**Figure 2:**
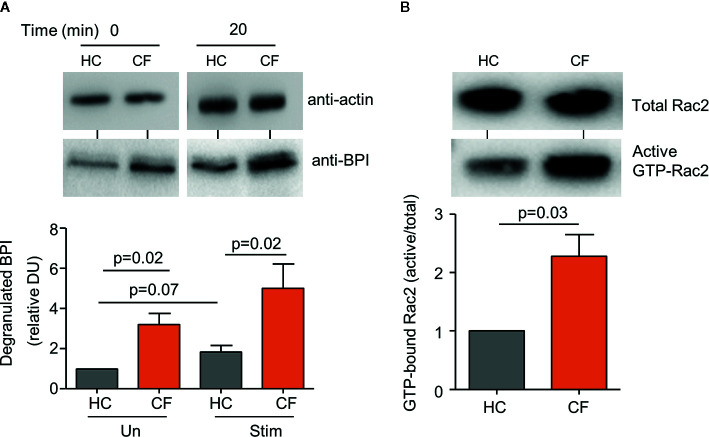
Increased degranulation of BPI from CF neutrophils. **(A)** Neutrophils from *Phe508del* homozygous PWCF (CF) or healthy controls (HC) remained unstimulated (Un) or were stimulated (Stim) with TNF-α (1 ng )/fMLP (100 ng) (2 × 10^7^ cells/mL) for 20 min and extracellular supernatants immunoblotted for degranulated BPI. Values presented as densitometry units (DU) and normalized to time zero of unstimulated HC cells. Levels of BPI released from unstimulated and stimulated CF cells was significantly greater than that of HC (p=0.02, n=5 subjects per group, Student’s t test). **(B)** Active GTP bound Rac2 (GTP-Rac2) was extracted from whole cell lysates of CF and HC neutrophils. GTP-Rac2 immuno-bands were normalized to total Rac2 levels and the HC value and expressed as a % of total Rac2. Significantly increased levels of GTP-Rac2 were detected in CF samples (p=0.03, Student’s t test, n=5 subjects per group). In **(A)** immuno-blots of whole cell lysates probed for actin determined equal protein loading, confirming equal cell numbers per reaction and representative blot images are shown. Each measurement is the mean ± SEM from biological replicates.

Previous studies have revealed a disparity in CF neutrophil degranulation of primary granule components including increased release of myeloperoxidase (MPO) and neutrophil elastase (NE) (Koller et al., 1995; Taggart et al., 2000). Following the finding of increased levels of BPI in plasma and BAL of PWCF, the level of BPI released by CF neutrophils was compared to HC cells. Within this set of experiments, a combination of TNF-α (1 ng) and fMLP (100 ng) was the chosen stimulus to trigger granule release. In unstimulated, and upon activation, release of BPI from primary granules was detected in the extracellular supernatant by immunoblotting and protein levels evaluated by densitometry. The level of BPI released by unstimulated CF neutrophils (p=0.02), and after 20 min stimulation (p=0.02), was significantly greater than HC cells (**Figure 2A**). The Rho GTPase Rac2 has been implicated in control of primary granule degranulation (Abdel-Latif et al., 2004), and therefore the levels of GTP-bound Rac2 in CF neutrophils was compared to healthy control cells. Using a GST-PAK-CRIB isolation method, GTP-bound Rac2 was precipitated from whole cell lysates, with subsequent total and active GTP-bound Rac2 content detected by immunoblotting. Results revealed a significant 2.5-fold increase in the level of GTP-bound Rac2 in unstimulated CF neutrophils compared to control cells (p=0.03) (**Figure 2B**). Collectively, these results illustrate increased Rac2 activation and increased secretion of BPI by CF cells, and thus the cause for impaired BPI activity in the CF airways was next explored.

### The Bactericidal Action of BPI Is Increased at Low pH and Unaffected by High Glycosaminoglycan Content of the CF Airways

**Figure 3:**
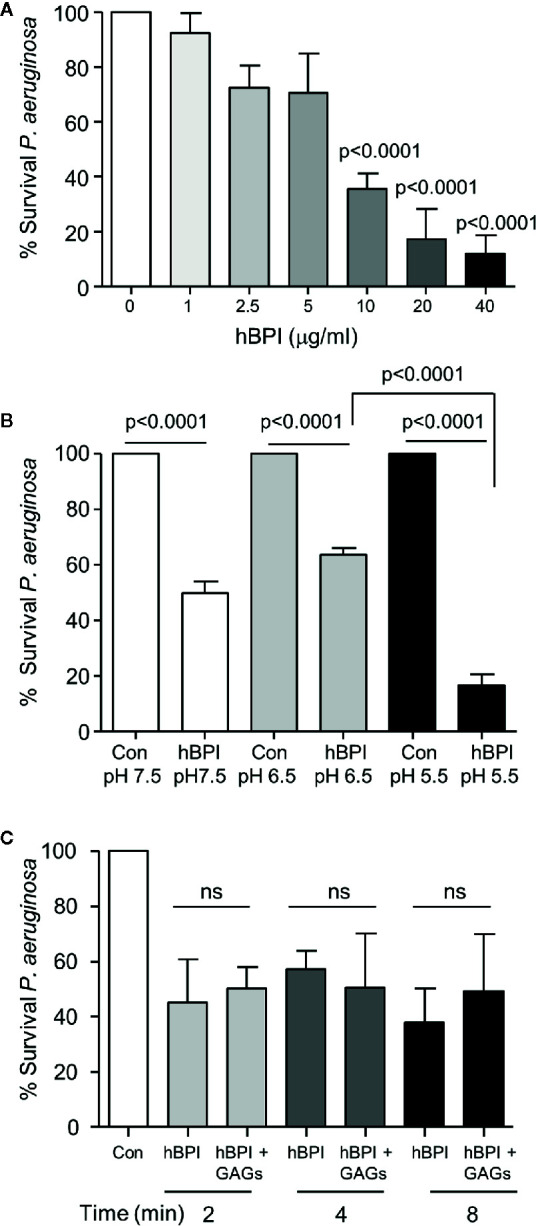
Bactericidal activity of BPI is unaffected by the pH and GAGs present in the CF airways. **(A)** Survival of *P. aeruginosa* (1 × 10^8^ c.f.u./ml) after 20 min incubation with BPI (1-40μg/ml). Data expressed as percentage survival of the untreated bacterial count. Bacterial survival was significantly decreased following treatment with 10, 20 or 40 μg/ml BPI (n=4 separate experiments, p<0.0001, One-way ANOVA test, followed by Bonferroni post-hoc test for selected groups). **(B)** To determine the effect of pH on the bactericidal effect of BPI, *P. aeruginosa* (1 × 10^8^ c.f.u./ml) was incubated at 37^°^C in 0·01 M phosphate buffer pH 7.5, 6·5 or 5·5 with or without BPI (10 µg/ml). Reduction in survival of *P. aeruginosa* by BPI at pH 5·5 compared to 6·5 was found to be significant (n=4 separate experiments, p<0.0001, two-way ANOVA test followed by Tukeys’ post-hoc test on unadjusted cfu data). **(C)** Bactericidal effect of BPI in the presence or absence of GAGs was determined by suspending *P. aeruginosa* (1 × 10^8^ c.f.u./ml) in phosphate buffer (pH 7·5) with BPI (10 µg/ml) with or without GAGs (1:10 (w/w) ratio) for 2, 4, or 8 min. No significant difference (ns) was recorded at any time point tested (n=3, two-way ANOVA test, followed by Tukeys’ post-hoc test on unadjusted cfu data).

The potent cytotoxicity of BPI is confined to gram negative bacteria (Elsbach and Weiss, 1993). As BPI protein was identified within the CF airways, and all recruited patients were colonized by *P. aeruginosa*, we explored parameters that may impede the antibacterial effect of BPI against *Pseudomonas* in the CF airways. In initial *in vitro* experiments, bacteria were exposed to increasing concentrations of BPI (1 to 40 µg/ml). A statistically significant reduction in survival was visible upon exposure to the physiologically relevant CF BAL concentration of 10 µg/ml BPI (p<0.0001) (**Figure 3A**), thus consolidating the need to understand the cause for impaired BPI killing of *P. aeruginosa* *in vivo*.

Studies in humans and CF cell lines indicate that pH of ASL is reduced in the absence of CFTR function (Smith and Welsh, 1992; Coakley et al., 2003; Abou Alaiwa et al., 2014a), and the more acidic pH diminished the effect of ASL antimicrobials including LL-37 and β-defensin-3 (Abou Alaiwa et al., 2014b). In contrast, results of the current study demonstrate that BPI (10 µg/ml) caused a significant reduction in *P. aeruginosa* survival at pH 7.5, 6.5 and 5.5, with the greatest reduction (~20% survival) observed at pH 5.5 (p<0.0001) (**Figure 3B**). Moreover, through interactions with negatively charged glycosaminoglycans (GAGs) present within the CF lung, it has been demonstrated that the antimicrobial activity of LL-37 is inactivated (Bergsson et al., 2009). In the current study, however, pre-exposure of BPI (10μg/ml) to a mixture of GAGs including heparan sulfate, chondroitin sulfate, and hyaluronic acid used at a 1:10 (w:w) ratio had no effect on BPI-induced bacterial killing across a time course of 2- 8 min (**Figure 3C**). Taken together, these data suggest that the antipseudomonal effect of BPI is unperturbed by factors known to inactivate antimicrobials in the CF airways.

### IgG Autoantibodies Present in the CF Airways Inhibit BPI Antimicrobial Activity

**Figure 4:**
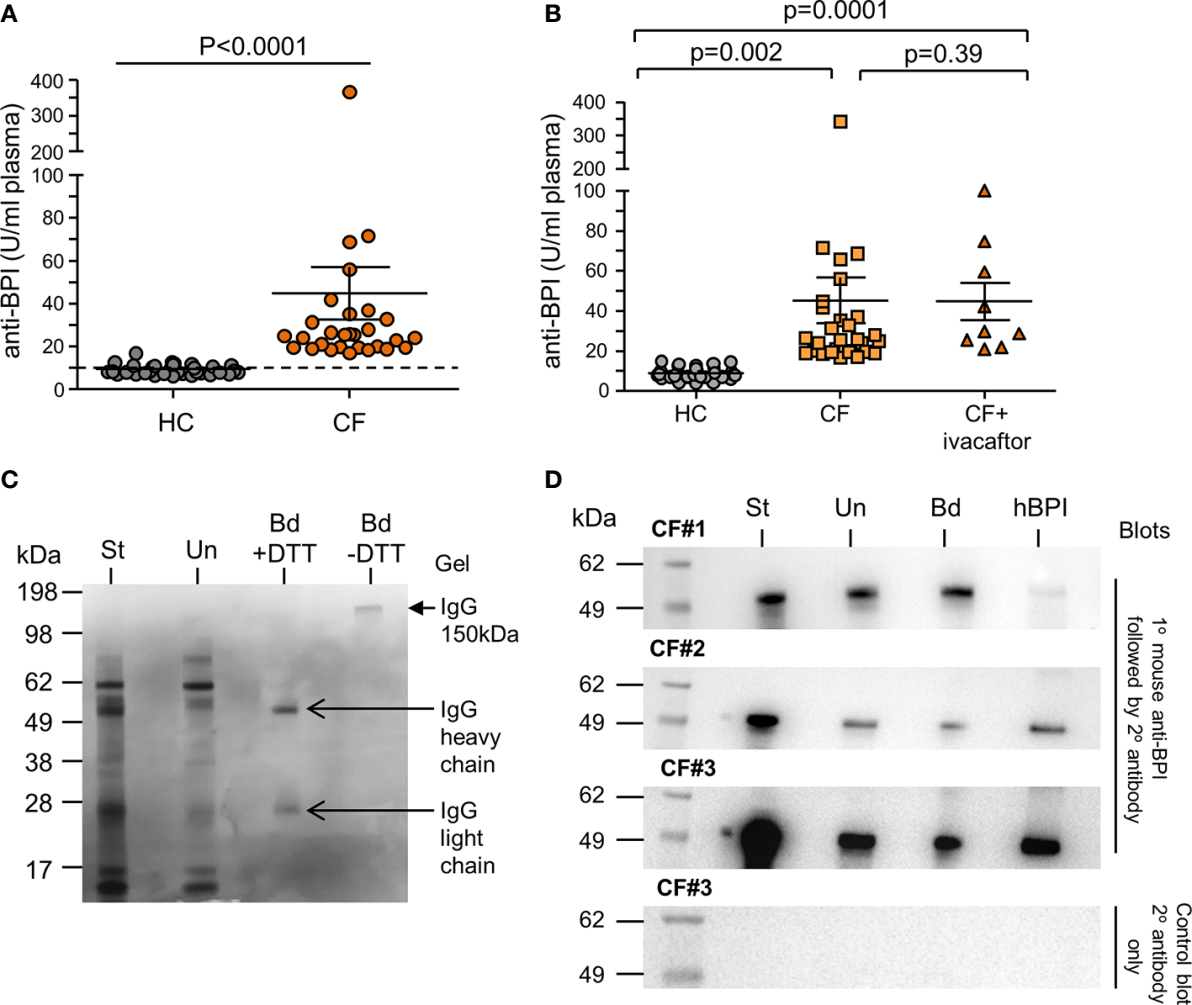
Increased levels of plasma autoantibodies and IgG-bound BPI in CF airway samples. **(A)** IgG autoantibodies against BPI were quantified in plasma of PWCF (CF, n=30) or healthy controls (HC, n=37). A significant increase in the titre of BPI autoantibodies were detected in CF (p<0.0001, Mann-Whitney U test). Positivity was set as 10 U/ml as indicated by the hatched line. All CF samples were positive for BPI autoantibodies. Increased circulating IgG BPI antibody level in CF individuals determined by ELISA. **(B)** Plasma samples from HC (n=38), CF (n=28) and CF individuals receiving ivacaftor treatment (n=10). No difference in levels of circulating anti-BPI IgG autoantibodies between CF and CF individuals receiving ivacaftor treatment (p=0.39). **(C)** Representative Coomassie blue stained SDS gel of purified IgG from CF BAL. Protein G Sepharose was used to isolate IgG from BAL of *Phe508del* homozygous PWCF. Starting BAL sample (St), unbound material (Un) or purified bound IgG (Bd) with or without DTT reduction are presented. Purified IgG (150 kDa, closed arrow), and the heavy (50 kDa) and light chains (25 kDa) of IgG are indicated (open arrows) (1 representative images of n = 5 biological repeats). **(D)** Protein G Sepharose was used to isolate IgG-BPI complexes from CF BAL. Reactions were analyzed by immunoblotting for BPI positivity using a mouse monoclonal anti-BPI antibody. Starting BAL sample (St), unbound material (Un) or IgG-bound BPI (Bd) are presented. Human BPI (hBPI) was used as a positive control (55 kDa). A control immunoblot to ensure BPI specificity omitted primary antibody (right hand panel), with no immune-band apparent. 3 representative images of n=5 biological repeats.

As a consequence of increased neutrophil degranulation and high plasma levels of BPI, the potential for the development of autoantibodies in adult PWCF was evaluated. A comparison of anti-BPI IgG-class autoantibodies in plasma from PWCF and HC was performed (**Figure 4A**). By employing a predetermined threshold level for positivity previously described as 10 U/ml, all 28 PWCF tested proved positive for autoantibodies against BPI, with recorded levels significantly higher than that observed for HC donors (p<0.0001). Using ELISA, we evaluated the effect of ivacaftor therapy on plasma levels of anti-BPI autoantibodies. Results revealed that the levels of anti-BPI autoantibodies in plasma of PWCF heterozygous for the *Gly551As*p mutation who were receiving ivacaftor for 2 years was significantly increased compared to HC (p=0.0001) but similar to PWCF not receiving therapy (p=0.39) (**Figure 4B**), thus confirming ongoing inflammation. Increased anti-BPI autoantibodies in plasma of PWCF is in line with previous reports (Zhao et al., 1996; Mahadeva et al., 1999), but to date, the presence with subsequent consequence of anti-BPI IgG antibodies in the CF lung has not been fully studied. Thus to first confirm that IgG antibodies are in CF airway samples, protein G sepharose was employed which has a high binding capacity for human IgG, but not IgA. Post incubation of protein G sepharose with CF BAL, immuno-precipitated protein was subjected to SDS-PAGE. The 150-kDa IgG protein was confirmed by Coomassie blue staining, which showed a single band under nonreducing conditions and two bands corresponding to the heavy and light chains after reduction by inclusion of DTT (**Figure 4C**).

To evaluate whether IgG-autoantibodies are present cross-linked to BPI in the CF lung, protein G sepharose precipitated reactions were subjected to Western blot analysis and probed for BPI. The antibody used for Western blotting was a mouse monoclonal antibody directed against residues 227-254, which links the *N-* (1-229) and *C-*terminal domain (251-456) of BPI. For each Western blot the starting BAL sample prior to the addition of protein G sepharose (St), the unbound protein material (Un), and the protein that bound the G sepharose is shown (Bd) (**Figure 4C**). Human BPI (hBPI) was used as a positive control (55 kDa). Immuno-band signals of the correct size positively identified BPI in reactions of protein G sepharose bound IgG from CF BAL (Bd fractions) (**Figure 4D**, 3 of 5 biological repeats presented). Positive signal in unbound fraction may represent free BPI or BPI bound to IgA ANCA, the latter known to be present in CF (Theprungsirikul et al., 2020), while the lesser signal intensity of bound and unbound fractions with respect to starting materials is a probable result of loss during wash steps. Hence, we have employed this setup as a qualitative approach. A control immunoblot to ensure BPI specificity omitted primary antibody (bottom panel). Collectively, this set of experiments indicates that neutrophil-released BPI is bound to IgG in the CF airways.

**Figure 5:**
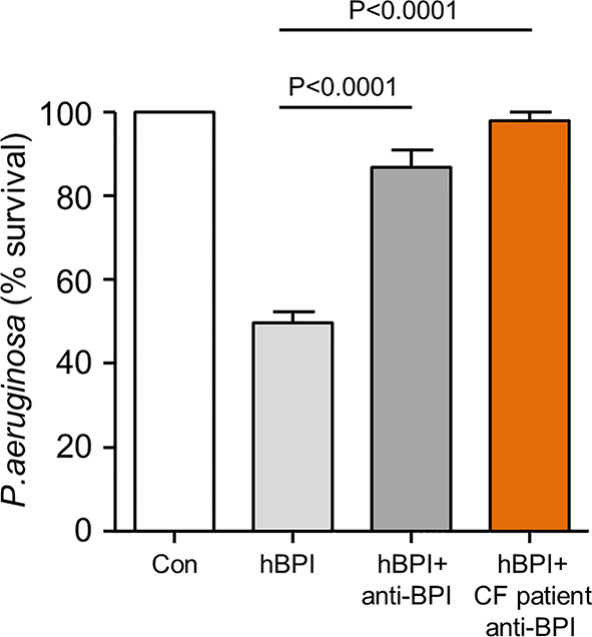
Autoantibodies in CF airway samples are associated with decreased BPI bacterial killing. Survival of *P. aeruginosa* (1 × 10^8^ c.f.u./ml) after incubation with BPI (10 μg/ml) in the presence or absence of 10μg/ml patient purified BPI autoantibodies or commercial anti-BPI antibody. Bacterial killing was significantly inhibited by CF BPI autoantibodies (p<0.0001, n = 4 separate experiments, One-way ANOVA test, followed by Bonferroni post-hoc test for selected groups).

Next, the possibility that autoantibody cross-linking of antigen could prevent BPI-induced *Pseudomonas* killing was examined. A bactericidal assay was carried out using 10 µg/ml of BPI in the presence and absence of equimolar anti-BPI antibody. The reduction in survival visible upon exposure of bacteria to BPI was significantly inhibited by inclusion of a commercially available *N*-terminal directed anti-BPI antibody (**Figure 5**). In addition, experiments exploring the effect of anti-BPI autoantibodies purified from CF samples revealed that purified autoantibodies mediated inactivation of BPI, resulting in a significant 2.5-fold increase in bacterial survival (p<0.0001) (**Figure 5**). From this set of experiments, we conclude that IgG class autoantibodies directed against BPI present in airway samples of PWCF, can target antigen and inhibit BPI bacterial killing.

## Discussion

Airway neutrophilia is common in PWCF and recruited neutrophils release large quantities of antimicrobial proteins and peptides from their cytoplasmic granules into surrounding delicate lung tissues. Moreover, *P. aeruginosa* dwelling in the airway can secrete factors that antagonise neutrophils, such as rhamnolipid or pyocyanin, that promote release of antimicrobials through direct lysis or induction of cell death (Allen et al., 2005; Jensen et al., 2007). Released antimicrobials are not fully effective at microbial clearance, and these granule proteins accrue to an extent that prompts the loss of self-tolerance and development of ANCA. BPI is present in the CF airway at high levels compared to healthy airways, and BPI-ANCA are present in the airways of a large proportion of people with CF (Schinke et al., 2004). In this study, we demonstrate that IgG class autoantibodies against BPI are antigen complexed in the CF lung, thereby inhibiting BPI anti-*Pseudomonas* cytotoxic activity. We additionally demonstrate that CF adults retain BPI ANCA seropositivity for at least two years following commencement on therapy with the CFTR modulator, ivacaftor.

Despite nomenclature describing their antigens as cytoplasmic, it is striking that the set of common ANCA antigens are each components of neutrophil primary granules, with MPO, proteinase 3 (PR3) and BPI being the most commonly described. CF neutrophils have been shown to have impaired degranulation processes, as greater levels of primary granule components including NE (Taggart et al., 2000) and MPO (Koller et al., 1995) were identified in the extracellular environment following stimulation of circulating CF cells. Excessive MPO levels have positively correlated with airflow obstruction and sputum production in *Phe508del* homozygous CF patients (Garner et al., 2004), in addition to augmenting oxidative damage to epithelial cells (Cantin et al., 1987; Cantin and Woods, 1993). Moreover, NE released upon dysregulated degranulation is the major destructive protease in the CF lung causing break down of the structural proteins, elastin, collagen and proteoglycans (Janoff et al., 1979; Cavarra et al., 1996). For this reason, the release of primary granules is tightly controlled by the small GTP-binding protein Rac2 (Abdel-Latif et al., 2004). In the current study increased GTP-bound Rac2 activation was observed, controlling BPI degranulation, leading to increased levels of extracellular BPI in both plasma and airway samples of PWCF. Despite this however, elevated BPI does not translate to clearance of *P. aeruginosa*. *In vitro*, we and others have demonstrated that *P. aeruginosa* is susceptible to BPI killing. We utilized strain PA01, a useful target microbe in this context as its innate resistance to BPI is high, attributable to *Pseudomonas* elastase (Skopelja et al., 2016). While *P. aeruginosa* can adapt to the CF airway over time, this does not explain the ability of environmental isolates to colonise people with CF, nor does it account for the observation that clinical, even mucoid, isolates of *P. aeruginosa* remain susceptible to BPI *ex vivo* (Aichele et al., 2006). Moreover, conditions that prevail in the CF lung milieu including low pH (Tate et al., 2002), and high concentrations of GAGs (Reeves et al., 2011), do not impair the cytotoxic effect of BPI, indeed, low pH supports increased BPI activity, as has previously been reported (Mannion et al., 1990). To our knowledge, the effect of BPI against *P. aeruginosa* present in an established biofilm is underexplored. We have shown BPI to be effective when combined in a mixture of glycosaminoglycans. Inasmuch as there is a superficial similarity between *Pseudomonas* alginate and our studied glycosaminoglycans, particularly their polyanionic character, it could be possible that BPI interacting with alginate would remain effective. Such positive antimicrobial effects of BPI supported early investigations into the potential therapeutic use of a recombinant amino-terminal fragment of human BPI in treatment of meningococcaemia (Giroir et al., 1997), and a randomized trial in children with severe meningococcal sepsis (Levin et al., 2000). These results and published observations raised the fundamental question of why BPI, present in high concentrations in the CF airway, fails to kill invading bacteria.

We have demonstrated that a proportion of extracellular BPI in the CF airway exists complexed to IgG autoantibodies. Incidence of BPI-directed ANCA in CF is particularly high in comparison to ANCAs against other neutrophil primary granule proteins such as MPO or NE, considering that these proteins are more abundant in neutrophil primary granules than BPI. For example, BPI ANCA positivity greatly exceeds that of PR3 ANCA in CF (Lachenal et al., 2009). The opsonizing capability of BPI may mean it is present in phagolysosomes during the disintegration of microbes that results in antigen presentation. It has also been proposed to result from the ability of BPI to deliver LPS-bacterial blebs to dendritic cells, thereby serving as a link between innate and adaptive immunity and a greater likelihood of self-reactive neo-epitope formation during antigen processing (Schultz et al., 2007). Our results demonstrate the presence of IgG class BPI ANCA in CF BAL samples. This result contrasts previous studies demonstrating fragmentation of IgG respiratory opsonins in the CF airways (Fick et al., 1984) and the identification of IgA rather than IgG in CF BAL (Theprungsirikul et al., 2020). An explanation for this disparity may in part be provided by the use of an alternative purification approach involving Protein G Sepharose in the current study, with full length IgG successfully identified in CF BAL.

Although studies have reported an increased incidence of BPI autoantibodies in PWCF for some time now (Zhao et al., 1996; Mahadeva et al., 1999), their actual role in inhibiting BPI mediated killing has received mixed reports. Some studies suggest that the BPI-ANCA present in the circulation are primarily raised against the C- terminal domain of the protein and thus do not inhibit killing (Dunn et al., 1999; Schultz et al., 2000), while others have noted that purified CF ANCA do in fact inhibit its bactericidal action (Sediva et al., 2003; Schultz et al., 2004). Our data illustrate the diminished antimicrobial potency of BPI against *P. aeruginosa* when complexed to IgG-class autoantibodies. Moreover, our data demonstrate that high levels of LPS present in CF BAL correlate with disease severity, and this raises the query of the consequence of BPI : IgG complexation. To further explain this point, BPI binding of LPS impedes delivery to CD14, thereby reducing immune cell activation (Marra et al., 1990; Leeuwenberg et al., 1994). Further studies are required to understand whether this anti-inflammatory role of BPI may possibly be impacted upon by IgG autoantibodies in the CF lung. To summarize, airway inflammation is exacerbated in PWCF through pro-inflammatory signaling resulting from higher bacterial burden and IgG-BPI immune complexes. As such, there is an unmet therapeutic need to disrupt these immune complexes or to limit the development of such antibodies.

In other disease settings where ANCA positivity is prominent, such as granulomatosis with polyangiitis, therapy with corticosteroids and rituximab are common, and reduce immune exertion and circulating B cell numbers, respectively. Indeed, a number of biologics targeting immune cells or their secreted components continue to be trialed in such conditions with some success (Basu et al., 2018). However, these ANCA-associated conditions do not co-present with chronic infection, and immunosuppressive therapy in chronically infected PWCF may not be advisable (King and Harper, 2017). Therefore, a targeted approach to abate BPI-ANCA development or function in CF would promote the endogenous antimicrobial BPI activity without off-target effects against the immune system.

One such tactic could include the use of small molecules. At the most basic level, provision of exogenous BPI to the airway could overwhelm ANCA interference while providing an additional antimicrobial effect. Such an approach might suit as adjunct therapy during pulmonary exacerbations, where the need for antibiotics is greatest. However, the potency of BPI when administered to patients who produce high levels of neutralizing anti-BPI antibodies needs to be explored. Our data show that, while BPI immune complexes exist in the airway, there may also be free BPI. Therefore, the protein is not immediately disabled. However, repeated dosing with BPI could also enhance BPI-ANCA production, diminishing BPI potency and exacerbating immune complex-mediated inflammation. Potentially, this may be alleviated by the use of small molecule decoys, peptidomimetics of BPI structural motifs, to neutralise the corresponding ANCAs (Vanpatten et al., 2016).

A second approach could include the use of anti-idiotype antibodies. The formation of antibodies that themselves target antibodies is a strategy that could be exploited to lessen the effect of ANCAs in CF. That said, the cost and complexity of developing a polyclonal anti-idiotype antibody against ANCAs could be prohibitive (Kohler et al., 2019). A third approach could include tolerogenic dendritic cell replacement therapy. Recent advancements in our understanding of the regulatory complexity of the immune system have enabled us to exert finer control over induction of specific components. Utilisation of tolerogenic dendritic cells (DCs) provide a highly relevant example. Pre-clinical work has shown that such cells can be programmed to suppress the function of T cells that would otherwise raise adaptive immunity against individual antigens such as BPI or MPO. Mice instilled with MPO-presenting tolerogenic DCs showed markedly reduced proliferation of MPO-specific T cells and, consequently, lesser glomerular injury (Odobasic et al., 2019). Extrapolation of this approach to PWCF could allow for suppression of the patients’ T cell contingent that promotes an anti-BPI adaptive response. DC therapy in CF has not been explored clinically. Pre-clinical investigations have previously shown that murine dendritic cells, exposed to *P. aeruginosa*, could be used to immunise recipient mice (Worgall et al., 2001), however, this approach has not been pursued in patients.

Looking to the future, we have entered an era of CFTR potentiators and correctors that can restore much of the function of the majority of CFTR variants. With improved airflow and normalized airway surface liquid composition, inflammation is reduced and airway remodeling minimized. Triple therapy of CFTR modulators – elexacaftor, tezacaftor and ivacaftor (together, Trikafta) – has recently been reported to provide significant improvements to airway function and extension of exacerbation-free periods of PWCF, further improving that provided by dual therapy (Middleton et al., 2019). Interestingly, our study found no significant difference in levels of anti-BPI autoantibodies in plasma of adult PWCF with the *Gly551As*p mutation receiving ivacaftor therapy compared to PWCF with different mutations. In this regard, we compared anti-BPI seropositivity in PWCF (N=28) who were corrector treatment naïve with those who had been receiving ivacaftor for the preceding two years (N=10). Prior to treatment, lung function was comparable between cohorts (FEV_1_ 42 ± 22 vs 53 ± 25, respectively). The continued presence of autoantibodies in adult PWCF with the *Gly551As*p mutation receiving ivacaftor therapy would suggest that the problem lies, not with CFTR dysfunction, but with the underlying irreversible chronically infected and inflamed bronchiectatic airways present in our CF adult cohort. As approval for CF modulator therapies continues to be granted to younger cohorts, who will receive these treatments before the onset of structural changes in their airways (Ridley and Condren, 2020) this may be less a problem in the future. In such cohorts, it is possible that BPI ANCA will not emerge, as airway inflammatory burden will not manifest to the same extent. Regarding late juvenile or adult PWCF in whom bronchiectatic changes are already established, there is an important gap in our knowledge: following long-term administration of corrector therapy, along with anti-inflammatories, whether the titre of BPI ANCA (IgA or IgG) may decline is unclear. This metric will be important to assess considering the future of *Pseudomonas* in CF.

It has been shown that ANCA titres lower during resolution of infection in non-CF settings (Ohtami et al., 2001). Accordingly, it is possible that if *P. aeruginosa* can be eradicated from the airways of PWCF that ANCA titres would lower, thereby reducing immune complex-mediated inflammation. The use of BPI as a therapeutic may be hindered if it is not effective at killing biofilm dwelling *P. aeruginosa*, hence strategies to improve biofilm disruption and *P. aeruginosa* eradication remain crucial. More broadly, BPI levels and titres of BPI ANCA reflect an overall severity of disease. We have previously introduced a combined metric, the CF-ABLE score, to codify this severity with respect to prognosis of death or transplant (Mccarthy et al., 2013). Through its use in clinical research, airway fluid NE and IL-1β have been shown to associate linearly with poor FEV_1_ and at the same time with worsening CF-ABLE score (Mcelvaney et al., 2019a; Mcelvaney et al., 2019b). It is known that higher BPI-ANCA titres correlate with lower lung function in PWCF with chronic *P. aeruginosa* infection (Carlsson et al., 2003). Further evidence is provided by a prospective 10-year study of whether IgA-BPI-ANCA positivity associates with prognosis in *P. aeruginosa*-infected CF patients (Lindberg et al., 2012). This study demonstrated that risk of mortality after 10 years is substantially greater in patients that have IgA-BPI-ANCA. Therefore, it is possible that a CF-ABLE score above 5, which itself predicts death or transplant in 4 years, would associate with high titres of BPI-ANCA.

This is an exciting field of investigation, and with the use of ivacaftor now from 6 months of age and the newest CFTR modulator combination therapy Trikafta for PWCF aged 12 years, it will be of major interest to discern whether these individuals will develop autoantibodies against products of activated neutrophils as they move from childhood to adolescence, and adolescence to adulthood. Equally, as measures to control chronic inflammation in CF develop, it will be important to understand whether this can confer a seronegative status on ANCA positive patients.

## Data Availability Statement

The raw data supporting the conclusions of this article will be made available by the authors, without undue reservation.

## Ethics Statement

The studies involving human participants were reviewed and approved by Ethical approval was received from the Beaumont Hospital Ethics Board (REC reference # 14/98) and informed consent obtained from all study participants. The patients/participants provided their written informed consent to participate in this study.

## Author Contributions

KM, FG, MM and OM performed experiments, analyzed and interpreted the data. ER, KM, MM and NM are responsible for study design and wrote the manuscript.

## Funding

In support of this work, ER acknowledges funding from the Medical Research Charities Group/Health Research Board Ireland.

## Conflict of Interest

The authors declare that the research was conducted in the absence of any commercial or financial relationships that could be construed as a potential conflict of interest.
